# Quantifying the dosimetric effects of neck contour changes and setup errors on the spinal cord in patients with nasopharyngeal carcinoma: establishing a rapid estimation method

**DOI:** 10.1093/jrr/rrac009

**Published:** 2022-04-03

**Authors:** Yinghui Li, Zhanfu Wei, Zhibin Liu, Jianjian Teng, Yuanzhi Chang, Qiuying Xie, Liwen Zhang, Jinping Shi, Lixin Chen

**Affiliations:** State Key Laboratory of Oncology in South China, Sun Yat-sen University Cancer Center, Sun Yat-Sen University of Medical Sciences, Guangzhou, 510060, Guangdong, China; Physics Department of the Radiotherapy Department, The First People’s Hospital of FoShan (Affiliated FoShan Hospital of Sun Yat-sen University), Foshan, 528000, Guangdong, China; Radiotherapy Center of the Oncology Medical Center, The First People’s Hospital of ZhaoQing, Zhaoqing, 526000, Guangdong, China; Physics Department of the Radiotherapy Department, The First People’s Hospital of FoShan (Affiliated FoShan Hospital of Sun Yat-sen University), Foshan, 528000, Guangdong, China; Physics Department of the Radiotherapy Department, The First People’s Hospital of FoShan (Affiliated FoShan Hospital of Sun Yat-sen University), Foshan, 528000, Guangdong, China; Physics Department of the Radiotherapy Department, The First People’s Hospital of FoShan (Affiliated FoShan Hospital of Sun Yat-sen University), Foshan, 528000, Guangdong, China; Physics Department of the Radiotherapy Department, The First People’s Hospital of FoShan (Affiliated FoShan Hospital of Sun Yat-sen University), Foshan, 528000, Guangdong, China; Physics Department of the Radiotherapy Department, The First People’s Hospital of FoShan (Affiliated FoShan Hospital of Sun Yat-sen University), Foshan, 528000, Guangdong, China; Physics Department of the Radiotherapy Department, The First People’s Hospital of FoShan (Affiliated FoShan Hospital of Sun Yat-sen University), Foshan, 528000, Guangdong, China; State Key Laboratory of Oncology in South China, Sun Yat-sen University Cancer Center, Sun Yat-Sen University of Medical Sciences, Guangzhou, 510060, Guangdong, China

**Keywords:** nasopharyngeal carcinoma (NPC), spinal cord (SC), dosimetric effects, setup error, contour change

## Abstract

The purpose of this study was to quantify the effect of neck contour changes and setup errors on spinal cord (SC) doses during the treatment of nasopharyngeal carcinoma (NPC) and to establish a rapid dose estimation method. The setup errors and contour changes in 60 cone-beam computed tomography (CBCT) images of 10 NPC patients were analysed in different regions of the neck (C1–C3, C4–C5 and C6–C7). The actual delivered dose to the SC was calculated using the CBCT images, and univariate simulations were performed using the planning CT to evaluate the dose effects of each factor, and an index ${\mathrm{Dmax}}_{\mathrm{displaced}}$ was introduced to estimate the SC dose. Compared with the planned dose, the mean (maximum) Dmax increases in the C1–C3, C4–C5 and C6–C7 regions of the SC were 2.1% (12.3%), 1.8% (8.2%) and 2.5% (9.2%), respectively. The simulation results showed that the effects of setup error in the C1–C3, C4–C5 and C6–C7 regions were 1.5% (9.7%), 0.9% (8.2%) and 1.3% (6.3%), respectively, and the effects of contour change were 0.4% (1.7%), 0.7% (2.5%) and 1.5% (4.9%), respectively. The linear regression model can be used to estimate the dose effect of contour changes (R^2^ > 0.975) and setup errors (R^2^ = 0.989). Setup errors may lead to a significant increase in the SC dose in some patients. This study established a rapid dose estimation method, which is of great significance for the daily dose evaluation and the adaptive re-planning trigger of the SC.

## INTRODUCTION

Nasopharyngeal carcinoma (NPC) is a very common malignant tumour disease in southern China, and radiotherapy is one of the main treatment modalities. NPC usually exhibits significant geometric uncertainties (contour changes and setup errors) during radiotherapy. These uncertainties may result in significant differences between delivered and planned radiotherapy doses [1, 2], which increases the risk of overdose exposure to organs at risk (OARs) [3–5]. Due to the mobility of the cervical spinal cord (SC), the geometric uncertainties of the neck are more significant than those of the head [6], and the dose safety of the SC itself has also received attention.

Several studies have reported the effects of these geometric uncertainties on the SC dose. Belshaw *et al.* [7], by rescan computed tomography (CT), found that the median increase in SC maximum dose was 0.7 Gy (range 0.2–1.9 Gy) but that this value increased to 7.2 Gy in a few patients. Noble *et al.* [8] reported on 133 patients with daily mega-voltage CT images, of whom 61 (45.9%) had delivered SC doses higher than the planned dose, and the difference in four patients was greater than 2 Gy. These studies concluded that there was no significant difference between the delivered SC dose and the planned SC dose. However, inconsistent conclusions have been reported in other studies. Han *et al.* [9] reported that the SC maximum dose showed a significant increase (3.3% to 15.5%) without daily setup corrections. Duma *et al.* [10] reported that the median increase in the delivered SC maximum dose compared with the planned dose was 10% for once weekly image-guided radiotherapy (IGRT) or non-IGRT. These studies recommend daily IGRT to eliminate a significant increase in the delivered SC dose. Even so, some studies have reported that the delivered maximum SC dose differed up to 13% after IGRT correction [11].

The inconsistent conclusions discussed above lead to confusion about whether it is necessary to adjust the treatment plan for SCs when there are significant geometric uncertainties in patients’ neck sites. In addition, almost all the studies have reported that there is a significant increase in the SC dose in some individual patients (approximately 3–10%) [7–8]; however, none of these studies further analysed the causes of significant dose differences in these patients.

To address the above confusion, we quantitatively evaluated the dosimetric effects of neck setup errors and contour changes on the SC during treatment and tried to establish a rapid evaluation method based on correlation analysis. First, we evaluated the delivered dose to the SC during treatment through cone-beam computed tomography (CBCT) images, which have been reported to be useful for accurate dose calculation [12–17]. Then, we used the planning CT to perform a single-factor simulation analysis on the actual setup errors and contour changes.

## METHODS AND MATERIALS

### Patients and CBCT images

Ten NPC patients, six males and four females with a median age of 46 years, who underwent volumetric-modulated arc therapy (VMAT) were selected for this study. Six patients were in cancer stage T3, four patients were in stage T4, and all the patients received docetaxel, cisplatin, and fluorouracil (TPF) induction chemotherapy. The prescribed dose of radiotherapy was 54 Gy to the planning target volume (PTV; PTV54), 62 Gy to the PTV62, 68 Gy to the PTV of the neck lymph node (PTVnd68) and 70 Gy to the PTV of the nasopharynx (PTVnx70) in 33 fractions. The dose constraint for the SC was set to a maximum dose of 40 Gy (Dmax, 0.1 cc), and the SC plus 5 mm margin, serving as the planning OAR volume (SC-PRV), was set to 45 Gy. Dose calculation was performed with the Eclipse v13.5 treatment planning system (TPS) (Varian Medical Systems, Palo Alto, CA) using an anisotropic analytical algorithm (AAA) and a 2.5 mm grid size.

The patients were treated by using a Varian Trilogy linear accelerator (Varian, Palo Alto, CA, USA) and were immobilized with a combination of a head rest, thermoplastic head and neck mask and carbon fibre base plate. The CBCT images were acquired with the Varian onboard imager (OBI) kV-CBCT system. The scanning parameters were 100 kV, 10 mA, and 20 ms (low-dose head mode), and the reconstruction volume was 512 × 512 with a resolution of 0.49 × 0.49 × 2.5 mm^3^. All patients were performed weekly CBCT scans (CBCT 1, CBCT 10, CBCT 17, CBCT 23, CBCT 28, and CBCT 33), and a total of 60 groups of CBCT data were used in this study.

### Setup errors and contour changes

All the CBCT images were manually and rigidly registered to the planning CT images based on soft tissue and bony structures by a single observer using the Eclipse registration module. Considering the differences in mobility in different regions of the SC [6], we divided the SC into three regions for setup error evaluation: cervical vertebrae 1–3 (C1–C3), cervical vertebrae 4–5 (C4–C5), and cervical vertebrae 6–7 (C6–C7). Each region was registered based on the bone structure of the cervical spine, and the setup error results were compared with the head results, which were mainly registered based on the nasal septum and pterygoid process.

**Fig. 1.**
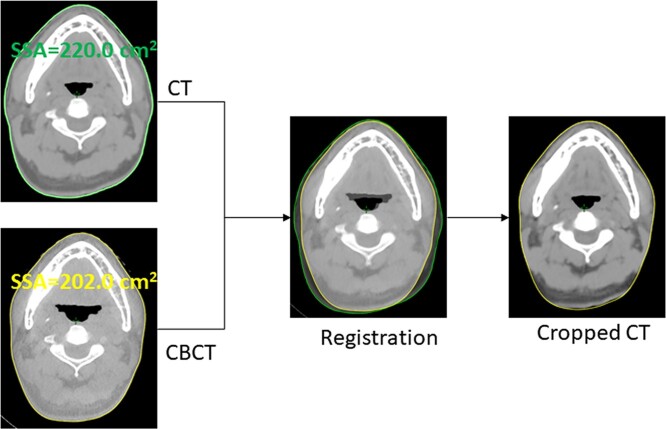
Schematic diagram of CT image clipping.

The setup errors in the left–right (x), inferior–superior (y) and anterior–posterior (z) directions were documented for different parts of the SC, and the changes in 3D setup errors ($\sqrt{\Delta{x}^2+\Delta{y}^2+\Delta{z}^2}$) during the treatment course were also evaluated. To evaluate the changes in neck contours during treatment course, the median contour (slice) surface area (SSA) [8] (Fig. 1) in each region of the SC (C1–C3, C4–C5 and C6–C7) was documented and used to evaluate the contour changes. The contour change was expressed as follows: $( SS{A}_{ct}- SS{A}_{cbct})/ SS{A}_{ct}$.

### Dose calculation and simulation

The actual delivered dose was calculated using the CBCT images, where the CBCT images were corrected for image quality using a U-net convolution neural network method [18]. The U-net network is an encoder-decoder structure, where the encoder gradually reduces the number of spatial dimensions and identifies the features of the image, while the decoder gradually repairs the details and spatial dimensions of the object and determines the boundary of the object pixel by pixel. After the U-net process, the difference of SC Dmax between CBCT and CT decreased from (0.3 ± 0.1) % to (0.1 ± 0.1) % [18].

To evaluate the effects of setup errors and contour changes on the SC dose, we simulated the single-factor effect of setup error or contour change on the SC dose distribution by adjusting the planning CT. To do this, the simulation for setup error was to adjust the iso-centre parameters according to the documented setup error in each region of the SC; the simulation for contour change was to clip the planning CT external contours according to the parameter of the CBCT external contour, as shown in Fig. 1. A total of 360 groups of dose distributions were calculated in this study, including 60 groups of planned doses, 60 groups of delivered doses, 180 groups of simulated doses for setup errors (60 groups for C1–C3, C4–C5 and C6–C7 respectively), and 60 groups of simulated doses for contour changes.

The dose of each treatment fraction was converted into the total prescription dose for evaluation, and the Dmax in each region of the SC was documented. The delivered Dmax, the planned Dmax, the setup error simulated Dmax and the contour change simulated Dmax were represented as ${\mathrm{Dmax}}_{\mathrm{delivered}}$, ${\mathrm{Dmax}}_{\mathrm{planned}}$, ${\mathrm{Dmax}}_{\mathrm{setup}}$ and ${\mathrm{Dmax}}_{\mathrm{contour}}$, respectively.

### Statistical analysis and dose estimation

The statistical significance (Wilcoxon signed rank sum test, *P* < 0.05) of the setup errors between the head (where the primary lesion and CT marker are located) and the SC regions in three directions was first analysed using IBM SPSS Statistics 20 software (SPSS Inc. Chicago, IL) and then evaluated the trend of the neck setup errors (3D) and contour changes with the treatment fractions. The percentage dose differences of the ${\mathrm{Dmax}}_{\mathrm{delivered}}$, ${\mathrm{Dmax}}_{\mathrm{setup}}$ and ${\mathrm{Dmax}}_{\mathrm{contour}}$ relative to the ${\mathrm{Dmax}}_{\mathrm{planned}}$ were quantified and visually compared using histograms.

To estimate the SC dose, we evaluated the relationships of the SC dose change with the setup errors and contour changes. The correlation of the contour changes was determined by fitting the SSA difference and the change in ${\mathrm{Dmax}}_{\mathrm{contour}}$ with a linear regression function. Due to the directionality of the setup errors, it is difficult to directly evaluate its effect on the SC dose. We assumed that the setup error only caused the displacement of the SC to the peripheral dose area and that the treatment dose distribution of the patient was not affected. To verify this hypothesis, we displaced the SC contour of the planning CT according to the setup error of each region of the SC. The Dmax of the SC after displacement was expressed as the ${\mathrm{Dmax}}_{\mathrm{displaced}}$, and the values were compared with the values of ${\mathrm{Dmax}}_{\mathrm{setup}}$ to evaluate whether the Dmax _displaced_ can be used as an index for estimating the SC dose.

## RESULTS

### Setup errors and contour changes

**Fig. 2.**
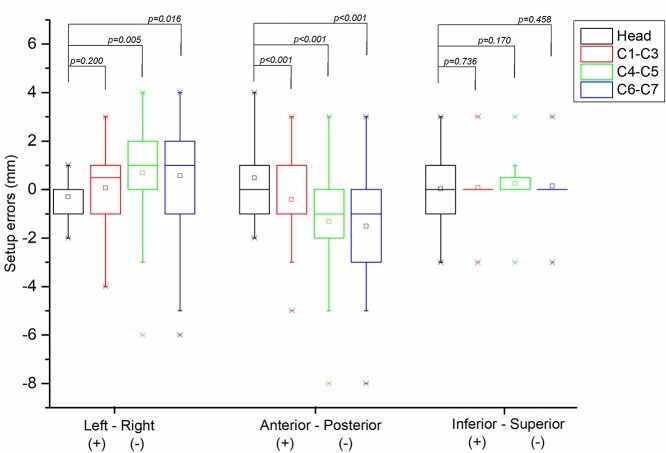
Analysis of the boxplot graph of setup errors in different parts in the left–right, anterior–posterior and inferior–superior directions.

The statistical results of the setup errors in the anterior–posterior, left–right and superior–inferior directions for the head and regions of the neck are represented by a boxplot graph, as shown in Fig. 2. In the anterior–posterior direction, the setup errors had statistical significance between the head and all regions of the neck (*P* < 0.001), the setup errors of the neck mainly tended to the posterior direction, and the maximum setup error was −8 mm. In the left–right direction, the setup errors of C4–C5 (*P* = 0.005) and C6–C7 (*P* = 0.016) had statistical significance with that of the head, and C1–C3 showed no statistical significance (*P* = 0.200). In the superior–inferior direction, no statistical significance in setup errors was found between the head and all regions of the neck (*P* = 0.736, 0.170 and 0.458 for C1–C3, C4–C5 and C6–C7).

The results of the 3D setup errors in the head and each evaluated region of the SC with the treatment fractions are shown in Table 1 and presented as the mean and standard deviation (mean ± SD). The setup errors of the head showed no significant change during the whole treatment course, with an average of 2.0 ± 0.9 mm. Compared with the results of the head, the setup errors of each region of the SC increased to varying degrees, including 2.4 ± 1.2 mm in C1–C3, 3.2 ± 1.8 mm in C4–C5 and 3.5 ± 1.8 mm in C6–C7. A significant increase mainly occurred in the C4–C5 and C6–C7 regions after the 23rd treatment fraction.

**Table 1:** The statistical results (mean ± SD) of the 3D setup errors for the head and regions of the neck

	Head Mean ± SD (mm)	C1–C3 Mean ± SD (mm)	C4–C5 Mean ± SD (mm)	C6–C7 Mean ± SD (mm)
CBCT1	1.8 ± 0.6	2.1 ± 0.4	2.4 ± 1.2	2.8 ± 0.9
CBCT10	2.1 ± 1.0	2.1 ± 1.3	2.5 ± 1.6	2.8 ± 1.4
CBCT17	1.9 ± 0.8	2.1 ± 1.0	2.7 ± 1.0	2.9 ± 1.3
CBCT23	2.2 ± 0.9	2.3 ± 1.5	4.0 ± 2.2	4.3 ± 2.2
CBCT28	2.0 ± 1.1	2.7 ± 0.9	3.9 ± 1.5	4.1 ± 1.6
CBCT33	2.1 ± 1.1	3.2 ± 1.3	4.0 ± 2.4	4.3 ± 2.3
Average	2.0 ± 0.9	2.4 ± 1.2	3.2 ± 1.8	3.5 ± 1.8

The contour changes with the treatment fraction in each region of the SC are shown in Table 2 and presented as the mean ± SD. During the whole treatment course, the contour changes in the C1–C3 region decreased from 192.7 cm^2^ to 179.8 cm^2^, those in the C4–C5 region decreased from 133.0 cm^2^ to 115.8 cm^2^ and those in the C6–C7 region decreased from 171.4 cm^2^ to 140.7 cm^2^. The average contour changes (reductions) in all the patients in the C1–C3, C4–C5 and C6–C7 regions were 3.6%, 6.7% and 9.1%, respectively.

**Table 2:** The statistical results (mean ± SD) for the median contour areas in each region of the neck

	C1–C3 Mean ± SD (cm^2^)	C4–C5 Mean ± SD (cm^2^)	C6–C7 Mean ± SD (cm^2^)

CT	192.7 ± 26.4	133.0 ± 28.8	171.4 ± 36.6
CBCT1	192.9 ± 27.1	133.9 ± 32.9	171.4 ± 37.5
CBCT10	189.1 ± 26.8	128.6 ± 30.5	164.2 ± 36.2
CBCT17	187.2 ± 25.6	127.1 ± 29.2	158.3 ± 31.1
CBCT23	184.0 ± 26.5	121.9 ± 28.4	151.6 ± 31.6
CBCT28	181.2 ± 25.4	118.3 ± 25.8	145.7 ± 32.1
CBCT33	179.8 ± 22.6	115.8 ± 22.7	140.7 ± 26.6
Average Change	(3.6 ± 4.0) %	(6.7 ± 6.7) %	(9.1 ± 8.6) %

### Dose comparisons

**Fig. 3.**
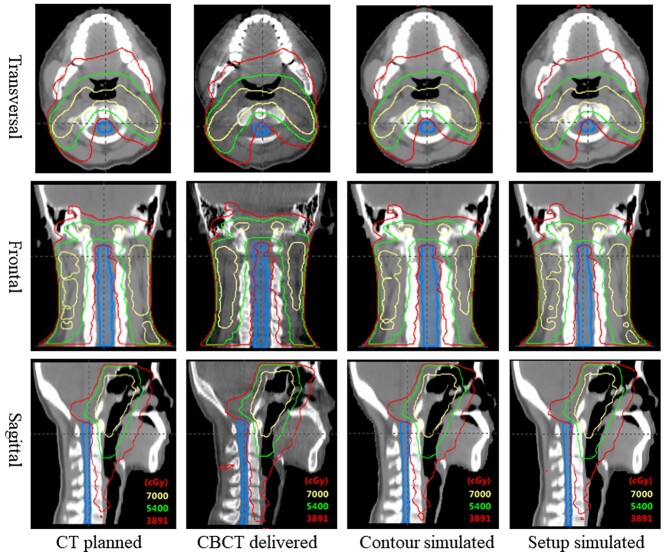
Dose distribution comparison of CT planned, CBCT delivered and the simulations of contour change and setup error (C1–C3). The blue line represents the SC, and the red, green and yellow isodose lines represent the ${\mathrm{Dmax}}_{\mathrm{planned}}$ (38.91 Gy), 54 Gy and 70 Gy, respectively.

Figure 3 shows side-by-side comparisons of the dose distribution between the planned, delivered, simulated contour change and simulated setup error (C1–C3) for a patient, the red isodose line represent the ${\mathrm{Dmax}}_{\mathrm{planned}}$ (38.91 Gy). Compared with the planned dose distribution, the red isodose line in the actual delivered dose distribution significantly invades the SC contour, especially the C1–C3 region; the simulation results showed that the contour change has no significant change in the dose distribution around the SC and that the setup error caused dose changes around C1–C3, similar to the delivered dose distribution.

**Fig. 4.**
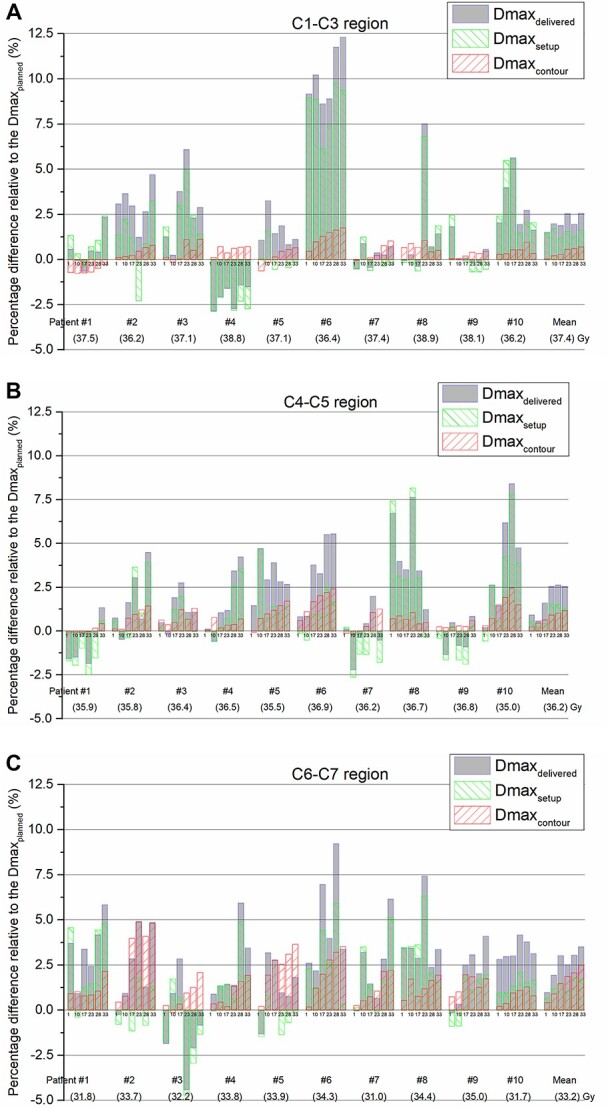
The percentage dose difference (relative to the planned Dmax) comparison among the ${\mathrm{Dmax}}_{\mathrm{delivered}}$, ${\mathrm{Dmax}}_{\mathrm{setup}}$ and ${\mathrm{Dmax}}_{\mathrm{contour}}$ in each region of the neck. (a) C1–C3, (b) C4–C5 and regions.

**Fig. 5.**
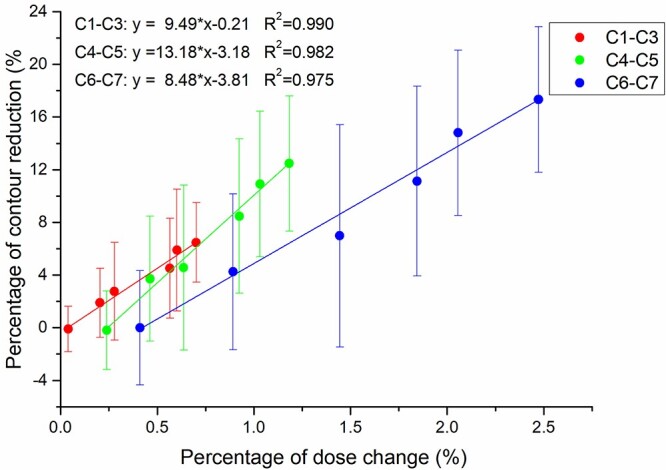
The relationship between the average neck contour change and average SC ${\mathrm{Dmax}}_{\mathrm{contour}}$ change in each region of the SC during the treatment course. The error bar represents the standard deviation of the contour changes. Red: C1–C3, Green: C4–C5, Blue: C6–C7.

Figure 4 shows the percentage dose differences in the ${\mathrm{Dmax}}_{\mathrm{delivered}}$, ${\mathrm{Dmax}}_{\mathrm{setup}}$ and ${\mathrm{Dmax}}_{\mathrm{contour}}$ relative to the ${\mathrm{Dmax}}_{\mathrm{planned}}$ for each region of the SC in 10 patients. The ${\mathrm{Dmax}}_{\mathrm{planned}}$ in the C1–C3 region was 37.4 Gy on average (range 36.2–38.9 Gy) and the Dmax point of the whole SC was mainly located in this region. Compared with the ${\mathrm{Dmax}}_{\mathrm{planned}}$ in C1–C3 region, the dose increase of the ${\mathrm{Dmax}}_{\mathrm{delivered}}$ was an average of 2.1% (range − 2.9% to 12.3%) (Fig. 4a). The effect of the setup error was an average of 1.5% (range − 2.8% to 9.7%), which was significantly larger than that of neck contour change (average 0.4%, range − 0.8% to 1.7%). Notably, patient #6 showed a significant dose difference in the C1–C3 region during the whole treatment course, the increase of ${\mathrm{Dmax}}_{\mathrm{delivered}}$ was 10.2% on average, range 8.6% to 12.3%). The ${\mathrm{Dmax}}_{\mathrm{planned}}$ in the C4–C5 region (Fig. 4b) was 36.2 Gy on average (range 35.0 to 36.9 Gy), and the dose increase in the ${\mathrm{Dmax}}_{\mathrm{delivered}}$ was 1.8% on average (range − 2.2% to 8.4%). Additionally, the setup error was the main reason for the dose increase (0.9% on average, range − 2.6% to 8.2%), and the effect of the neck contour change was only 0.7% on average (range − 0.2% to 2.5%). The ${\mathrm{Dmax}}_{\mathrm{planned}}$ in the C6–C7 region (Fig. 4c) was 33.2 Gy on average (range 31.0 to 35.0 Gy), and the dose increase in the ${\mathrm{Dmax}}_{\mathrm{delivered}}$ was 2.5% on average (range − 4.4% to 9.2%). The setup error and neck contour change, which were 1.3% on average (range − 4.7% to 6.3%) and 1.5% on average (range − 0.1% to 4.9%), respectively, had the same dose effect in the C6–C7 region.

### Dose estimations

A reduction in the neck contour will lead to an increase in the SC dose. As shown in Fig. 5, the Dmax change caused by the contour change can be evaluated by a linear regression equation. The data used for fitting were the average of all patients in each treatment fraction, and the correlation coefficients (R^2^) of the C1–C3, C4–C5 and C6–C7 regions were 0.990, 0.982 and 0.975, respectively. According to the results of the average contour changes in Table 2, it is easy to estimate that the average dose increases of C1–C3, C4–C5 and C6–C7 were 0.39%, 0.75% and 1.52%, respectively.

**Fig. 6.**
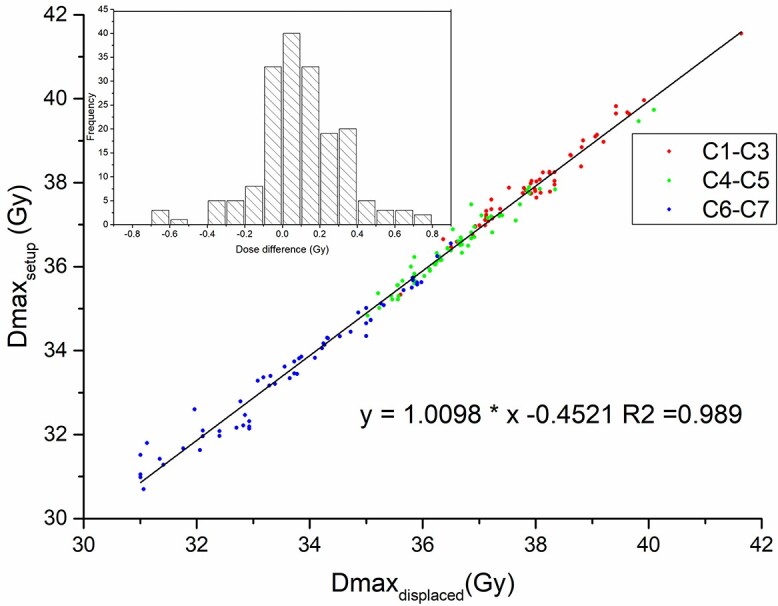
Correlation between the SC ${\mathrm{Dmax}}_{\mathrm{setup}}$ and the ${\mathrm{Dmax}}_{\mathrm{displaced}}$ in each evaluated region of the neck. Red: C1–C3, Green: C4–C5, Blue: C6–C7. The subgraph shows the difference between them.

The setup error may lead to an increase or decrease in the delivered dose relative to the planned dose, which is related to the offset direction. The ${\mathrm{Dmax}}_{\mathrm{displaced}}$ can well reflect the rise and fall in the delivered dose of the SC. As shown in Fig. 6, there was a strong linear relationship (R^2^ = 0.989) between the ${\mathrm{Dmax}}_{\mathrm{setup}}$ and the ${\mathrm{Dmax}}_{\mathrm{displaced}}$. The Dmax dose in any region of the SC can be estimated by the same fitting formula, and the dose deviation between them was only 0.1 ± 0.23 Gy, as shown in the subgraph in Fig. 6.

## DISCUSSION

In this study, we systematically analysed the setup errors and contour changes in the neck regions in NPC patients during radiotherapy, quantified the effects of these factors on the Dmax of the SC, and established a fast Dmax estimation method based on the correlations.

IGRT is the most common method for evaluating the anatomical changes in patients in the current clinical treatment. According to the report of Nabavizadeh *et al.* [19], 86% of head and neck patients were evaluated by daily IGRT, and 11 patients were evaluated by weekly IGRT. Nevertheless, Duma *et al.* [20] reported that despite daily IGRT, the delivered dose to the SC may still be significantly different from the planned dose. The primary goal of this study was to determine the main factor for the significant Dmax deviation in the SC in some patients. Through an intuitive quantitative comparison with simulation results, we found that the setup error is the main factor for the significant increase in the delivered Dmax in the C1–C3 (Fig. 4a) and C4–C5 (Fig. 4b) regions. Especially in the C1–C3 region (where the Dmax point of the SC is located), the maximum effect of contour changes was only 1.7%, while the maximum effect of setup errors was 9.7%. Notably, although the setup errors were the main factor for the large increase in the SC dose, the C4–C5 and C6–C7 regions with more serious setup errors did not exhibit a more significant dose change than the C1–C3 region. There are two main reasons [20] for this observation: first, the C4–C5 and C6–C7 regions are relatively far from the high-dose radiation target (PTV), so the surrounding dose gradients are relatively flat; second, due to the reduction in the soft tissue in the posterior neck, serious setup errors are located in the posterior direction (Fig. 2), so that the SCs are farther from the high radiation areas.

The second goal of this study was to establish a method to quickly estimate the SC dose during treatment. Noble *et al.* [8] first established a linear regression model to estimate the SC dose effect of contour changes but found that there was no correlation between contour changes and Dmax changes in the SC. By avoiding the interference of setup error (univariate simulation analysis), this study proved that there was a linear correlation between contour change and SC dose change (Fig. 5). Of course, due to the limitation of the small sample size, the standard deviation is large, and a more accurate correlation may need to be supported by a larger sample size. Similarly, we also performed univariate simulation on the setup error and proposed the index of the ${\mathrm{Dmax}}_{\mathrm{displaced}}$ to estimate the effect of setup errors on the SC dose. This method simplifies the complex 3D vector information of the setup errors to a univariate relationship (Fig. 6**)**.

The body shape changes in patients can be used as an adaptive re-planning trigger for OARs. For example, You *et al.* [21] demonstrated that adaptive planning can be useful for patients with significant anatomic contour changes (decrease in neck diameter [>10%]) to reduce xerostomia caused by the parotid gland. However, due to the weak correlation between contour changes and changes in the SC dose [8], significant body shape changes are not suitable as a trigger for SC re-planning, so it is recommended to be triggered by evaluating the SC dose [7]. Therefore, the method proposed in this study is a good supplement to the daily IGRT of the SC, which helps to trigger the clinical decision-making of adaptive re-planning.

There are many methods for evaluating the SC dose [7–11], such as the CBCT dose calculation method. The advantage of the proposed method is that it does not need additional contour delineation and dose calculation, and its operation almost does not take time and workload, which makes it feasible to carry out daily SC dose evaluation. Considering that NPC patients may have different setup errors and contour changes in other facilities, this study also proved that the proposed method is suitable for the Elekta synergy accelerator, as shown in the supporting information (Figs S1 and S2). This single disease is another limitation of this study and it will be further investigated whether the proposed method is applicable to other head and neck diseases.

## CONCLUSIONS

In this study, the dosimetric effects of neck geometric uncertainties on the SC during treatment were quantitatively analysed, and a fast method for estimating the actual delivered Dmax to the SC was proposed. Based on this method, we can quickly evaluate the daily dose to the SC on the basis of daily IGRT, which is of great significance to trigger the adaptive re-planning of the SC.

## CONFLICT OF INTEREST

The authors declare that they have no competing interests.

## FUNDING

This work was supported by the Medical Science and Technology Foundation of Guangdong Province (A2021186), Spacial fund of Foshan Summit plan (2019D009).

## Supplementary Material

Supporting_information_rrac009
