# Supplementary material for: Quantifying the dosimetric effects of neck contour changes and setup errors on the spinal cord in patients with nasopharyngeal carcinoma: establishing a rapid estimation method
Source: J Radiat Res. 2022 Apr 3;63(3):443–51. doi: 10.1093/jrr/rrac009 (PMC9124625; doi:10.1093/jrr/rrac009)
Supplement: Supporting_information_rrac009 [file supporting_information_rrac009.docx]

**S1 Fig.** The percentage dose difference (relative to the planned Dmax) comparison among the Dmax_delivered_, Dmax_setup_ and Dmax_contour_ of the SC in NPC patients treated by Elekta synergy accelerator.

Results presentation: The dose increase in the $\mathrm{Dmax}_{\mathrm{delivered}}$ was 3.5% on average (range -4.5% to 11.1%) relative to the $\mathrm{Dmax}_{\mathrm{planned}}$. The effect of the setup error was 2.6% on average (range -3.6% to 11.9%), which was significantly larger than that of the neck contour change (0.8% on average, range -1.0% to 2.4%), which was similar to Varian trilogy accelerator.





**S2 Fig.** Correlation between the SC $\mathrm{Dmax}_{\mathrm{setup}}$ and the $\mathrm{Dmax}_{\mathrm{displaced}}$ in NPC patients treated by Elekta synergy accelerator.

Results presentation: The SC $\mathrm{Dmax}_{\mathrm{setup}}$ can be estimated by the linear fitting formula, and the dose deviation between $\mathrm{Dmax}_{\mathrm{setup}}$ and $\mathrm{Dmax}_{\mathrm{displaced}}$ was 0.1±0.43 Gy

**Patients and CBCT information**

Fifteen NPC patients, namely, 8 males and 7 females, who underwent volumetric-modulated arc therapy (VMAT) were selected for this study, with a median age of 59 years. Ten patients were in cancer stage T3, 5 patients were in stage T4, and all the patients received docetaxel, cisplatin, and fluorouracil (TPF) induction chemotherapy. The prescribed dose of radiotherapy was 54 Gy to the planning target volume (PTV; PTV54), 66 Gy to the PTV of the neck lymph node (PTVnd66) and 70 Gy to the PTV of the nasopharynx (PTVnx70) in 33 fractions. The dose constraint for the SC was set to a maximum dose of 45 Gy (Dmax, 0.1 cc), and the SC plus 3 mm margin as the SC-PRV. Dose calculation was performed with the Monaco v5.11 treatment planning system (TPS) using the Monte Carlo algorithm and a 2.5 mm grid size.

The patients were treated by using a Elekta Synergy linear accelerator (Elekta, Crawley, UK) and the CBCT images were acquired with the Elekta XVI kV-CBCT system. Each patient underwent a CBCT scan (CBCT17) during treatment, and the scanning parameters were 100 kV, 36.6mAs, and the reconstruction volume was 270×270 with a resolution of 1.0×1.0×3 mm^3^.
